# Recycling tenofovir in second-line antiretroviral treatment with dolutegravir: outcomes and viral load trajectories to 72 weeks

**DOI:** 10.1097/QAI.0000000000003157

**Published:** 2023-04-15

**Authors:** Claire M Keene, Tali Cassidy, Ying Zhao, Rulan Griesel, Amanda Jackson, Kaneez Sayed, Zaayid Omar, Andrew Hill, Olina Ngwenya, Gert van Zyl, Tracy Flowers, Eric Goemaere, Gary Maartens, Graeme Meintjes

**Affiliations:** 1 Medecins Sans Frontiers, Cape Town, South Africa; 2 Health Systems Collaborative, Oxford Centre for Global Health Research, Nuffield Department of Medicine, University of Oxford; 3 Division of Public Health Medicine, School of Public Health and Family Medicine, University of Cape Town, Cape Town, South Africa; 4 Department of Medicine, University of Cape Town, Cape Town, South Africa; 5 Wellcome Centre for Infectious Diseases Research in Africa, Institute of Infectious Disease and Molecular Medicine, University of Cape Town, Cape Town, South Africa; 6 Division of Clinical Pharmacology, Department of Medicine, University of Cape Town, Cape Town, South Africa; 7 University of Liverpool, Department of Pharmacology, Liverpool, United Kingdom; 8 Stellenbosch University, Division of Medical Virology, Cape Town, South Africa and National Health Laboratory Service, Tygerberg Business Unit, Cape Town, South Africa

**Keywords:** TLD, dolutegravir, recycling-NRTIs, low-level viraemia, second-line antiretroviral therapy

## Abstract

**Background:**

Recycling tenofovir and lamivudine/emtricitabine with dolutegravir (TLD) after failure of non-nucleoside transcriptase inhibitor (NNRTI) first-line antiretroviral therapy (ART) is more tolerable and scalable than dolutegravir plus optimized nucleoside reverse transcriptase inhibitors. Studies have demonstrated TLD’s efficacy as second-line, but long term follow-up is limited.

**Methods:**

ARTIST is a single arm, prospective, interventional study conducted in Khayelitsha, South Africa, which switched 62 adults with two viral loads (VL) >1000 copies/mL from tenofovir, lamivudine/emtricitabine and an NNRTI to TLD. We report efficacy to 72 weeks and, in a post hoc analysis, evaluated VL trajectories of individuals with viraemic episodes.

**Results:**

Virologic suppression was 86% (95% Confidence Interval (CI) 74-93), 74% (95% CI 61-84) and 75% (95% CI 63-86) <50 copies/mL, and 95%, 84% and 77% <400 copies/mL at week 24, 48 and 72 respectively, with 89% (50/56) resistant (Stanford score ≥15) to tenofovir and/or lamivudine pre-switch. No participants developed integrase-inhibitor resistance. Of the 20 participants not suppressed at week 24 and/or 48, two developed virologic failure, one switched regimen (adverse event), two were lost to follow-up, one missed the visit, one transferred out, nine resuppressed <50 copies/mL with enhanced adherence counselling and four remained viraemic (three with <200 copies/mL) at week 72.

**Conclusions:**

Recycling NRTIs with dolutegravir was effective for most participants to 72 weeks. Most with viraemia did not develop virologic failure and subsequently suppressed with enhanced adherence counselling or continued to have low-level viraemia. No integrase-inhibitor resistance was detected despite low-level viraemia in a minority of participants.

## Introduction

The DAWNING study showed that dolutegravir was superior to ritonavir-boosted lopinavir after failing first-line non-nucleoside reverse transcriptase inhibitor (NNRTI)-based antiretroviral therapy (ART)^1^. In both arms, the backbone was dual nucleoside reverse transcriptase inhibitors (NRTI) with at least one NRTI fully active on resistance testing^1^, which led the World Health Organization to recommend a second-line regimen of zidovudine, lamivudine and dolutegravir (ALD) after failing a first-line NNRTI-based regimen with tenofovir plus lamivudine or emtricitabine (XTC) in settings with limited access to antiretroviral resistance testing^2^. This recommendation for the zidovudine and lamivudine NRTI backbone is based on the rationale that the resistance mutation selected for by tenofovir (K65R), does not compromise the activity of zidovudine^3^.

Recent evidence has emerged that recycling tenofovir and XTC with dolutegravir (TLD) in second-line ART, after failure of first-line NNRTI-based ART, is efficacious despite high rates of resistance to both tenofovir and XTC^4,5^. Such a strategy would be beneficial to both patients and the health system as tenofovir is better tolerated than zidovudine, and TLD is available as a single fixed dose tablet taken once daily while ALD requires twice daily dosing of zidovudine and lamivudine ^6^.

We have previously reported 24-week results of a single-arm interventional study of TLD as second-line ART (AntiRetroviral Therapy In Second-line: investigating Tenofovir-lamivudine-dolutegravir [ARTIST] trial), with 85% (51/60) achieving the primary end point of VL <50 copies/mL despite substantial baseline resistance to one or both of tenofovir and XTC (88.9%, 48/54). No participants developed study-defined virologic failure and no integrase resistance was detected in the one participant meeting criteria for resistance testing^4^.

The NADIA trial found similar results after randomising participants failing first-line ART consisting of an NNRTI, tenofovir, and XTC, first to darunavir or dolutegravir, and second to tenofovir or zidovudine with lamivudine. Dolutegravir was non-inferior to darunavir at 48 and 96 weeks^5,7^, and recycling tenofovir was non-inferior at 48 weeks^5^ and superior at 96 weeks^7^ to switching to zidovudine. This was achieved despite more than half of the participants having no fully active NRTI on resistance testing in the tenofovir arm^5^. In the VISEND trial, TLD or a regimen of dolutegravir with tenofovir alafenamide and emtricitabine, were both superior to boosted protease inhibitor regimens with zidovudine and lamivudine at week 48, with virologic suppression of 82%, 87% and 76% respectively^8^.

The efficacy of second-line TLD in the absence of a fully active NRTI is likely due to dolutegravir’s high genetic barrier to resistance coupled with a cost in fitness from NRTI resistance mutations^9^. However, the development of integrase inhibitor resistance has been described in clinical trials^1,7,10–12^ and post-market programmatic settings^13,14^. In the NADIA trial, three of the nine cases of treatment-emergent dolutegravir resistance occurred in the TLD group^7^. In a Malawian observational study following patients transitioning to TLD with a raised VL (n=101), dolutegravir resistance developed in two patients who had virologic failure on second-line TLD^14^.

Emergence of dolutegravir resistance in patients on second-line TLD could jeopardise the public health benefits that the combination potentially offers in lower and middle income country programmes. It is unclear whether the efficacy of TLD in those who have failed first-line NNRTI-based therapy is durable, particularly in those who develop viraemia, making longer follow-up crucial to inform treatment policy decisions^15^. Here we report follow-up results of the ARTIST trial participants to 72 weeks to evaluate the maintenance of the virologic suppression on second-line TLD. Additionally, we conducted a post hoc descriptive analysis of the VL trajectories of those who had episodes of viraemia.

## Methods

### Study design

We conducted a single arm, prospective, interventional study in two primary care clinics in Khayelitsha, South Africa. This study was designed to evaluate the virologic efficacy of TLD in second-line ART.

A full description of the study design was published with the week 24 primary endpoint results^4^. The protocol was approved by the University of Cape Town’s Human Research Ethics Committee (039/2019) and is available with the statistical analysis plan on ClinicalTrials.gov (NCT03991013). Adults who had virologic failure (defined as two consecutive VL >1000 copies/mL, 2-24 months apart) on a first-line regimen consisting of tenofovir, XTC and efavirenz or nevirapine were invited to participate. Exclusion criteria were CD4 cell count less than 100 cells/ml, active or suspected tuberculosis, active AIDS-defining conditions, an estimated glomerular filtration rate less than 50 ml/min per 1.73m^2^, haemoglobin less than 7.5 g/dl, alanine aminotransferase greater than 100 IU/l, a previous or current diagnosis of malignancy or any condition judged to put the patient at increased risk if participating, a condition judged likely to impact adherence (active psychiatric disease or substance abuse), pregnancy, breastfeeding, intention to fall pregnant or unable to take the study medication (allergy, intolerance or contraindicated drug interaction). Women of child-bearing potential were required to be on effective contraception.

Participants were enrolled and switched to TLD with additional 50 mg dolutegravir for two weeks to overcome efavirenz induction effects on dolutegravir metabolism and transport. Study visits with clinicians occurred every four weeks to week 24, then at week 36, 48 and 72. VL was assessed at baseline and every subsequent visit, with a repeat VL after two weeks of enhanced adherence counselling if VL was >50 copies/mL after week 12. If the repeat VL was >500 copies/mL, a genotypic resistance test was performed per protocol. Baseline genotypic resistance testing was performed retrospectively for all participants and was not available to inform treatment decisions.

### Outcomes and analysis

The primary outcome was VL suppression (defined as VL <50 copies/mL), evaluated according to the FDA snapshot algorithm^16^. We used a ± two-week visit window for the first 20 weeks, then a ± six-week visit window from week 24 onwards. The extended window was introduced to accommodate COVID-19 restrictions in accessing health facilities. We conducted a modified intention-to-treat analysis (mITT), excluding those switching study drug due to contraception cessation, wishing to become pregnant, pregnancy, non-clinical transfer out or death from non-HIV and non-drug causes (assessed by the study doctor). Failure included: VL ≥50 copies/mL, missing VL within the visit window or loss to follow up, intolerance or adverse event due to any drug in the regimen requiring switch.

Virological failure was defined as having two consecutive VL >1000 copies/mL after week 12. Genotypic resistance was classified using the Stanford algorithm (version 8.9-1), with a score ≥15 indicating at least low-level resistance. Results were categorised as two fully active NRTIs (both with a Stanford score <15), resistance to one NRTI (one with a Stanford score <15 and one ≥15) and resistance to both NRTIs (both with a Stanford score ≥15)^17^.

Virologic outcomes at week 24, 48 and 72 are presented as proportions with the corresponding exact binomial 95% confidence interval (CI). A pre-specified secondary analysis described VL suppression defined as <400 copies/mL. A sensitivity analysis of VL suppression excluded certain participants included in the mITT analysis: those lost to follow-up, missing a VL in the window and those who were changed from the study drug for reasons other than treatment failure. The pre-specified sensitivity analysis for the primary end-point of 24 weeks also excluded those with evidence of poor adherence at the visit (TFV-DP <350 fmol/punch), however these results were not available for the 48 and 72 week visits.

In a post hoc descriptive analysis, the VL trajectories of participants who were on TLD and had a VL ≥50 copies/mL at weeks 24 and/or 48 were graphed along with a narrative description of their baseline and follow-up resistance test results, reported adherence (taken from clinician notes) and missed visits (no visit within the window).

## Results

### Baseline characteristics

The baseline characteristics of the 62 participants enrolled between August 2019 and July 2020, are described in Table 1. Low, intermediate or high-level resistance to tenofovir, XTC or both was found in 89.3% (50/56 participants with results). Of these, 35.7% (20/56) had K65R mutations and 82.1% (46/56) had M184V/I mutations.

### Virologic outcomes at week 24, 48 and 72

The proportion of participants with virologic suppression (VL <50 copies/mL) decreased from week 24 to week 72 but was maintained above 70% across the mITT and sensitivity analyses. In the mITT analysis 75.4% of participants had a VL <50 copies/mL at 72 weeks. A sensitivity analysis examined virologic suppression in those with a VL result who remained on TLD throughout the study; at 72 weeks 83.6% had a VL <50 copies/mL in this analysis (Table 2).

The outcomes of those not suppressed at weeks 24, 48 and 72 are described in Table 3. At week 48, of the 16 participants not suppressed, 6/16 (38%), and 6/12 on TLD with VL results, had low level viraemia <200 copies/mL. At week 72, 5/15 (33%), and 5/9 of those on TLD with VL results, had VL <200 copies/mL.

### Virologic failure

Two participants met criteria for virologic failure (two consecutive VL >1000 copies/mL after week 12) during the follow up period: one at 36 weeks and one at 52 weeks. Three participants were eligible for resistance testing and all three had resistance testing conducted (including the two participants with virological failure), but none were found to have integrase resistance mutations.

### Suppression by baseline NRTI resistance

The relative risk of virologic suppression for those with any baseline NRTI resistance (Stanford score ≥15 for tenofovir, XTC or both^3^) compared to those with two active NRTIs at baseline was 0.84 (95% CI 0.74-0.95) for week 24, 0.91 (95% CI 0.61-1.34) for week 48 and 0.91 (85% CI 0.61-1.34) for week 72 (see Supplementary Figure 1 for proportion suppressed at each time point by baseline resistance to tenofovir, XTC or no resistance).

### Follow up of participants not suppressed at week 24 and/or week 48

Nine participants were not suppressed (VL <50 copies/mL) at week 24 (Table 3). Following these participants forward over time, one participant developed virologic failure, four re-suppressed, two had a VL <200 copies/mL and one was lost to follow-up by week 48. One had already switched from TLD before week 24 due to an adverse event (renal impairment). By week 72, three of those who had re-suppressed remained suppressed and one had a VL 500-999 copies/mL, one of the two with a VL <200 copies/mL remained viraemic (VL of 70 copies/mL) and one re-suppressed (Supplementary Figure 2).

**Figure 1:**
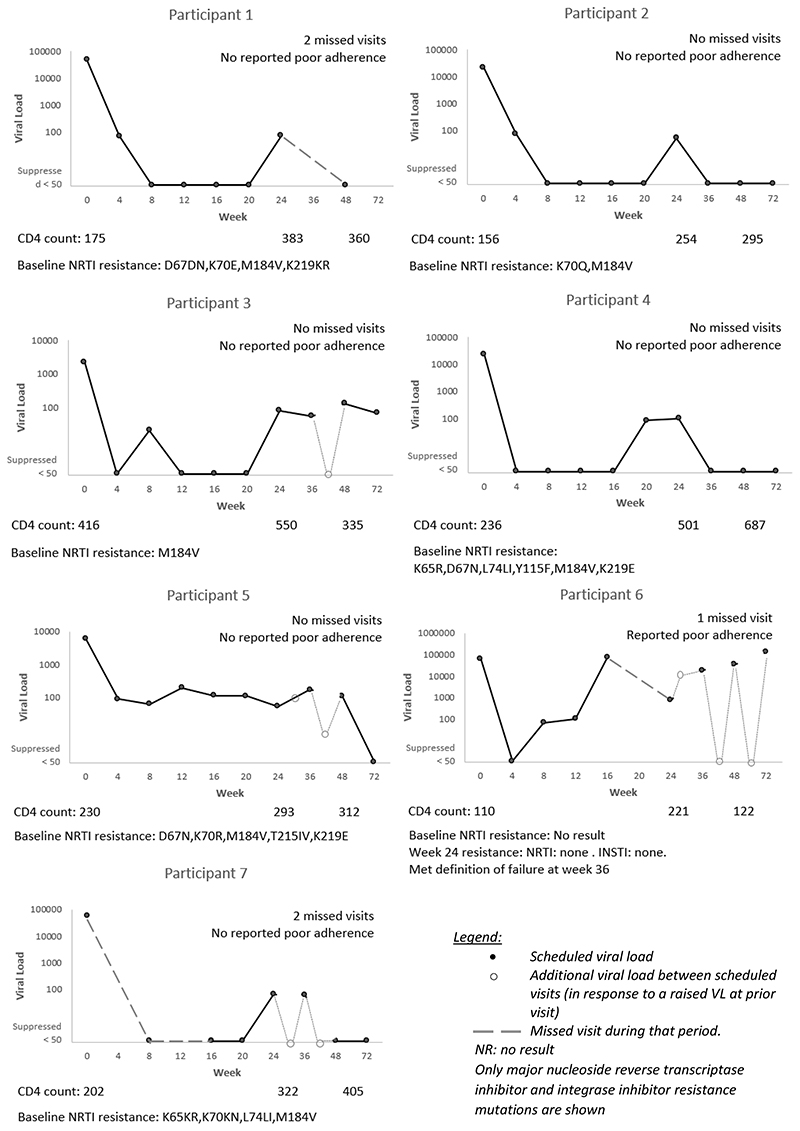
Individual trajectories of the seven participants on tenofovir, lamivudine and dolutegravir with a viral load result but not suppressed (VL ≥50 copies/mL) at week 24

We plotted the VL trajectories for participants who were on TLD, had a VL conducted and had a result ≥50 copies/mL at week 24 and/or week 48. Figure 1 describes the VL trajectories for the seven participants who had a VL ≥50 copies/mL at week 24, excluding the participant who was not on TLD because they had switched due to an adverse event, and the participant who was classified as not suppressed due to a missing VL result at week 24.

**Figure 2:**
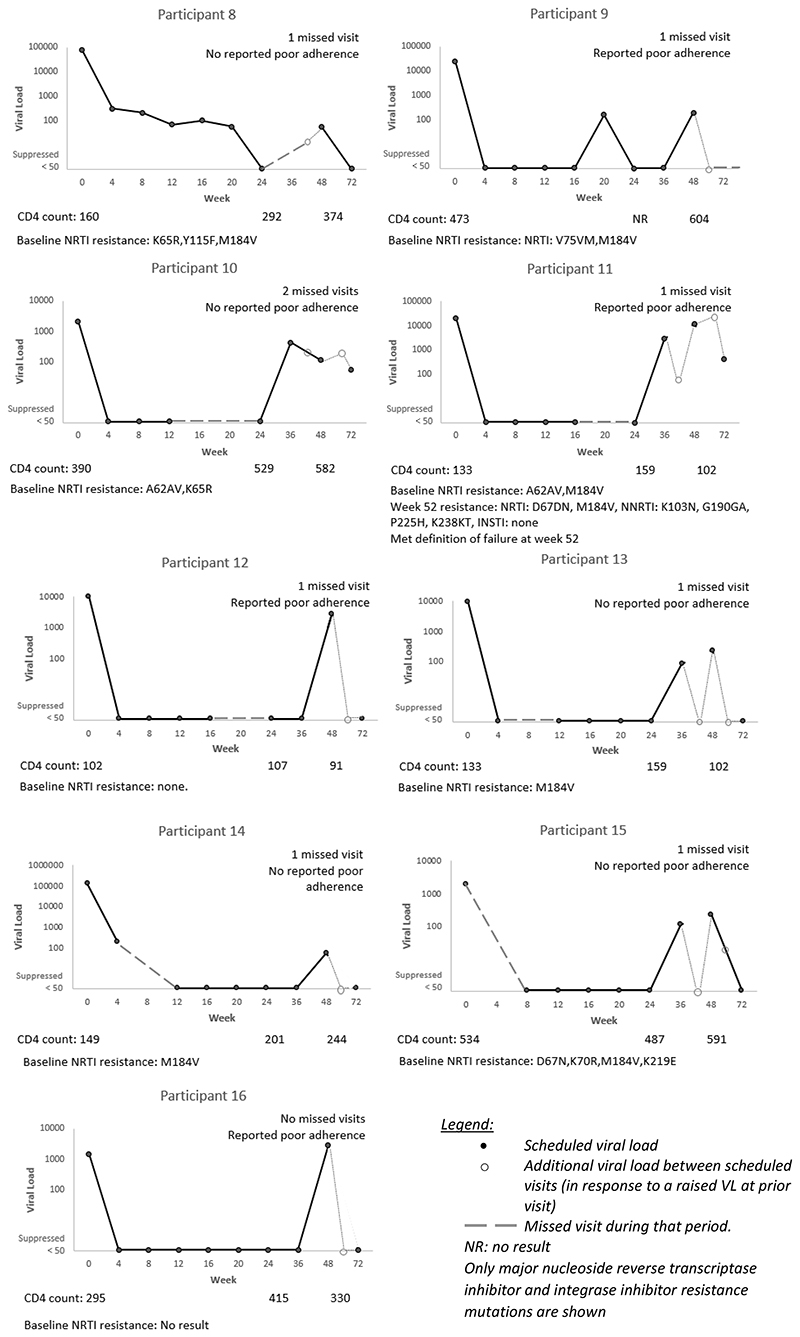
Individual trajectories of the nine participants on tenofovir, lamivudine and dolutegravir with a viral load result at week 48, who were suppressed at week 24 but not suppressed at week 48

Of the nine not suppressed at week 24, four were also not suppressed at week 48. Sixteen participants in total were not suppressed at week 48 (Table 3). By week 72, two had developed virologic failure, seven re-suppressed, three had a VL <200 copies/mL, one had switched regimen and three did not have a VL at week 72 (two of these were lost to follow up). Figure 2 describes the VL trajectories of the nine participants who had a VL ≥50 copies/mL at week 48 (excluding the five who were not suppressed at week 24 as they are described in Figure 1, and excluding two who were classified as not suppressed due to a missing VL result at week 48).

Overall, of the 20 participants who had not been suppressed at week 24 and/or week 48 (four at week 24 only, five at both time points, and 11 at week 48), six had a VL ≥50 copies/mL, three were missing VL results, one had switched due to an adverse event and ten were suppressed <50 copies/mL at week 72 (Supplementary Figure 2).

## Discussion

Our study demonstrated that virologic suppression is maintained above 70% to 72 weeks, despite almost 90% of participants having resistance to one or more NRTI drugs at baseline. The COVID-19 pandemic restricted access to facilities for many participants over the follow-up period: a sensitivity analysis that accounted for this and included only those on TLD with a VL result, also demonstrated high proportions with sustained virologic suppression: 88%, 79% and 84% with VL <50 copies/mL and 98%, 89% and 85% with VL <400 copies/mL at weeks 24, 48 and 72 respectively. Three quarters of the participants with episodes of viraemia re-suppressed to <50 copies/mL after enhanced adherence counselling, while two participants remained viraemic at low levels and only two went on to meet criteria for virological failure.

The outcomes we report are similar to the NADIA trial, where 92% were suppressed <400 copies/mL on a second-line regimen of dolutegravir or darunavir with a recycled tenofovir/lamivudine backbone at 48 and 96 weeks, and tenofovir/lamivudine was found to be superior to zidovudine/lamivudine at 96 weeks as a second-line backbone ^5,7^. Differences in suppression rates could be due to differences in patterns of adherence between the study populations. NADIA found suppression rates over 90% in those with no predicted NRTI activity^5^. We found a similar proportion suppressed in those with baseline NRTI resistance, and the Malawian observational study found no evidence for increased risk of viraemia or virological failure for participants with baseline NRTI resistance ^14^. This supports the conclusion from the NADIA trial that dolutegravir in combination with a recycled NRTI backbone is an effective second-line regimen. Most of our participants who were not suppressed had low-level viraemia (VL <200 copies/mL), and most of these resuppressed at later time points with enhanced adherence counselling. While there was little self-reported poor adherence, this may be due to a social desirability bias. Those with missed visits reflect gaps in medication on hand, leading to poor adherence. The NADIA trial also postulated that poor adherence was prevalent in their population^5^. We hypothesise that suboptimal adherence resulted in low-level viraemia and subsequent re-suppression with improved adherence.

Despite the substantial proportion with low-level viraemia, only two participants fulfilled the study definition of virological failure over 72 weeks and neither of these participants (nor an additional patient who qualified for genotype resistance testing) had integrase resistance mutations. Resistance has been identified in NADIA (three cases in the TLD group)^7^ and the Malawian observational study (two cases)^14^. The relatively low median baseline VL at switching and the small sample size in ARTIST may have contributed to our finding that no patients developed resistance to dolutegravir over 72 weeks. However, as resistance to dolutegravir has been shown in dolutegravir monotherapy trials to generally develop between 24 and 48 weeks^10,11^, these results are reassuring. This finding in participants using recycled tenofovir in the NRTI backbone, is important considering the high prevalence of tenofovir resistance in patients failing first-line ART in sub-Saharan Africa^18^. Cycling in and out of care with periods of viraemia is to be expected over a lifetime of ART ^19,20^. Thus, sustaining virologic suppression over time requires a regimen that is robust to the development of resistance despite fluctuating adherence. Dolutegravir has a particularly high barrier to resistance, comparable to protease inhibitors and much higher than efavirenz^21^. Low level viraemia has also been shown not to be associated with subsequent virologic failure in patients on a dolutegravir regimen, nearly half of whom had previous virologic failure^22^.

Dolutegravir with recycled tenofovir and lamivudine seems to fulfil this need for a tolerable, robust second-line regimen and could help in achieving the third UNAIDS target of 95% of those on treatment being virologically suppressed^23^. Low-level viraemia at the primary end-point did not predict later virologic failure in participants on first-line TLD in the ADVANCE study, resulting in a recommendation that the goal of treatment on dolutegravir regimens could be shifted to less stringent targets than a VL <50 copies/mL^24^. The low proportion of participants meeting criteria for virologic failure on TLD despite ongoing low-level viraemia in our study seems to support this recommendation.

The demonstrated efficacy of recycling tenofovir and lamivudine with dolutegravir in patients failing first-line NNRTI regimens in our study and the NADIA trial^4,7^ addresses two key concerns facing HIV services in this phase of the HIV pandemic: first, patients on second-line ART are in need of more tolerable regimens than the currently-recommended zidovudine/lamivudine/dolutegravir combination, and the fixed-dose, once-daily TLD provides a safe alternative to make treatment more acceptable and effective for individual patients.

Second, scale up of ART is complicated by resource constraints and many programmes are opting for universal TLD regimens, frequently without VL testing before switching patients due to high cost and low availability^25,26^. The growing evidence from this study, the NADIA trial, and other studies, suggests that switching all patients on NNRTI-based first line to TLD regardless of VL is effective and safe, with the caveat that monitoring and interventions to reduce the development of dolutegravir resistance should be implemented alongside widespread switching. This approach could facilitate less expensive and simpler strategies for wider TLD use ^21^.

Suboptimal adherence in a number of participants in this study did not result in virological failure with the development of integrase-inhibitor resistance mutations. This is compatible with dolutegravir’s high resistance barrier, though larger numbers and longer follow-up are needed to verify this. However, adherence challenges remain an issue regardless of regimen. While TLD represents a tolerable and robust second-line that may be helpful in improving adherence and outcomes in those who have previously failed treatment, individuals on this regimen will still benefit from adherence support^27^. Effective monitoring and management of adherence issues is required to sustain the population health benefits that TLD promises.

Limitations of this study include the higher proportion of missing VL results at week 48 and 72. COVID-19 reduced access to the primary care facilities where the study took place, and while we widened the visit window and used telephonic follow-up to mitigate this, the pandemic may still be responsible for missing VL data. Interpretation of our study is also limited by the small sample size, not having a control arm and lack of long-term therapeutic drug monitoring. While the frequent VL monitoring is not pragmatic for clinical practice, it allowed exploration of virologic trajectories following episodes of viraemia. A strength of our study is that it was embedded within a primary care ART clinical service, increasing generalisability

## Conclusion

Most of our participants on a regimen of dolutegravir with a recycled tenofovir and lamivudine NRTI backbone were virologically suppressed at 72 weeks, despite substantial baseline NRTI resistance. Most of those who did not suppress had low-level viraemia or missed visits. Only two participants with raised VL developed virologic failure, and no participants were found to have developed dolutegravir resistance. With enhanced adherence counselling most participants re-suppressed or continued to have low-level viraemia. These findings, together with those of the NADIA trial and other studies, support the routine switching of all patients on tenofovir and NNRTI-based first-line therapy to TLD regardless of viral load, alongside measures to mitigate and detect the development of dolutegravir resistance. This would simplify rollout and make this effective, tolerable, and inexpensive regimen available to millions more patients.

## Supplementary Material

Supplementary material

## Figures and Tables

**Table 1:** Baseline characteristics of all participants

Baseline characteristics, n=62	
**Participant characteristics**	
Female sex [n(%)]	43 (69%)
Age (years) [median(IQR)]	36.4 (30.9-46.0)
BMI (kg/m^2^) [median(IQR)]	27.8 (22.6-32.4)
Weight (kg) [median(IQR)]	71.2 (62.1-81.4)
**HIV characteristics**	
CD4 count (cells/μL) [median(IQR)]	259 (175-380)
VL (copies/mL) [median(IQR)]	9586 (2827-37243)
**ART history**	
ART duration (years) [median(IQR)]	5.2 (2.8-8.2)
Prior exposure to stavudine or zidovudine [n(%)]	10 (16%)
On efavirenz at enrolment [n(%)]	61 (98%)
Baseline genotypic resistance* **(n=56), [n(%)]**	
NRTI genotypic resistance	
Two fully active NRTIs	6/56 (11%)
Resistance to one NRTI	
- Tenofovir, not XTC	0/56 (0%)
- XTC, not tenofovir	14/56 (25%)
Resistance to both NRTIs	36/56 (64%)
Efavirenz and/or nevirapine genotypic resistance	54/56 (96%)

ART (antiretroviral therapy); IQR (inter-quartile range); NRTI (nucleoside reverse transcriptase inhibitor); VL (viral load); XTC (lamivudine or emtricitabine).

* Resistance classified using Stanford interpretation, where a score <15 indicates susceptible or potential low-level resistance to a drug, and ≥15 indicate low-level, intermediate, or high-level resistance to a drug. For this analysis resistance was defined as a score ≥15 ^17^

**Table 2:** Viral load outcomes at week 24, 48 and 72

	VL suppression <50copies/mL (n; percentage, 95% CI)		VL suppression <400copies/mL (n; percentage, 95% CI)	
	mITT analysis*	Sensitivity analysis**	mITT analysis*	Sensitivity analysis**
Week 24	53/62; 85 (74-93)	53/60; 88 (77-95)	59/62; 95 (87-99)	59/60; 98 (91-100)
Week 48	45/61; 74 (61-84)	45/57; 79 (66-89)	51/61; 84 (72-92)	51/57; 89 (78-96)
Week 72	46/61; 75 (63-86)	46/55 84 (71-92)	47/61; 77 (65-87)	47/55 85 (73-94)

* Modified intention-to-treat analysis (mITT) excludes those switching study drug for reasons of stopping contraception or wish to become pregnant, or becoming pregnant, transfer out for non-clinical reasons and death from non-HIV and non-drug causes.

** Sensitivity analysis excludes those excluded from mITT analysis, as well as those lost to follow up, those missing a VL within the window and participants who stopped or were changed from the study drug for reasons other than failure of the regimen

**Table 3:** Outcomes of those not suppressed at the week 24, 48 and 72 visits.

	Week 24	Week 48	Week 72
	N(%)	N(%)	N(%)
**Not suppressed**	9/62 (15%)	16/61 (26%)	15/61 (25%)
**Virologic failure (two consecutive VL >1000 copies/mL after 12 weeks)**	0/62 (0%)	1/61 (2%): failed at week 36	2/61 (3%): failed at week 36 and week 52
**VL > 1000 copies/mL but did not meet the definition of failure**	0/62 (0%)	3/61 (5%)	0/61 (0%)
**VL 400-999 copies/mL**	1/62 (2%)	0/61 (0%)	2/61 (3%)
**VL 200-399 copies/mL**	0/62 (0%)	2/61 (3%)	0/61 (0%)
**VL 100-199 copies/mL**	0/62 (0%)	4/61 (7%)	1/61 (2%)
**VL 50-99 copies/mL**	6/62 (10%)	2/61 (3%)	4/61 (7%)
**No VL data**	1/62 (2%): lost to follow-up at week 16	3/61 (5%): 1 missed a visit in the study window but still in the study, 2 lost to follow-up	5/61 (8%): 3 missed a visit in the study window but still in the study, 2 lost to follow-up
**Other**	1/62 (2%): switched due to an adverse event before 24 weeks	1/61 (2%): switched due to an adverse event before 24 weeks	
		*1 transferred out for non-clinical reasons at week 36, and so was not included in the analysis (reduced denominator to 61)*	
