# Supplementary material for: Recycling tenofovir in second-line antiretroviral treatment with dolutegravir: outcomes and viral load trajectories to 72 weeks
Source: J Acquir Immune Defic Syndr. Author manuscript; Available in PMC 2023 Apr 15. (PMC7614301; doi:10.1097/QAI.0000000000003157)
Supplement: Supplementary material [file EMS159082-supplement-Supplementary_material.pdf]

Supplementary material

Recycling tenofovir in second-line antiretroviral treatment with dolutegravir: outcomes and viral load trajectories to 72 weeks

Claire M KEENE[1,2], Tali CASSIDY[1,3], Ying ZHAO[4,5], Rulan GRIESEL[5,6], Amanda JACKSON[5], Kaneez SAYED[5], Zaayid OMAR[5], Andrew HILL[7], Olina NGWENYA[5], Gert VAN ZYL[8], Tracy FLOWERS[1], Eric GOEMAERE[1,3], Gary MAARTENS[5,6], Graeme MEINTJES[4,5]

[1]*Medecins Sans Frontiers, Cape Town, South Africa*

[2]*Health Systems Collaborative, Oxford Centre for Global Health Research, Nuffield Department of Medicine, University of Oxford*

[3] *Division of Public Health Medicine, School of Public Health and Family Medicine, University of Cape Town, Cape Town, South Africa*

[4]*Department of Medicine, University of Cape Town, Cape Town, South Africa*

[5]*Wellcome Centre for Infectious Diseases Research in Africa, Institute of Infectious Disease and Molecular Medicine, University of Cape Town, Cape Town, South Africa*

[6]*Division of Clinical Pharmacology, Department of Medicine, University of Cape Town, Cape Town, South Africa*

[7]*University of Liverpool, Department of Pharmacology, Liverpool, United Kingdom*

[8] *Stellenbosch University, Division of Medical Virology, Cape Town, South Africa and National Health Laboratory Service, Tygerberg Business Unit, Cape Town, South Africa.*

Supplementary material contents

- Figure 1. Proportion suppressed <50 copies/mL at each study visit in the modified intention-to-treat analysis using the Food and Drug Administration snapshot algorithm, categorised by baseline nucleoside reverse transcriptase inhibitor resistance

- Figure 2. Virologic outcome at each time point for those not suppressed at week 24 and/or week 48

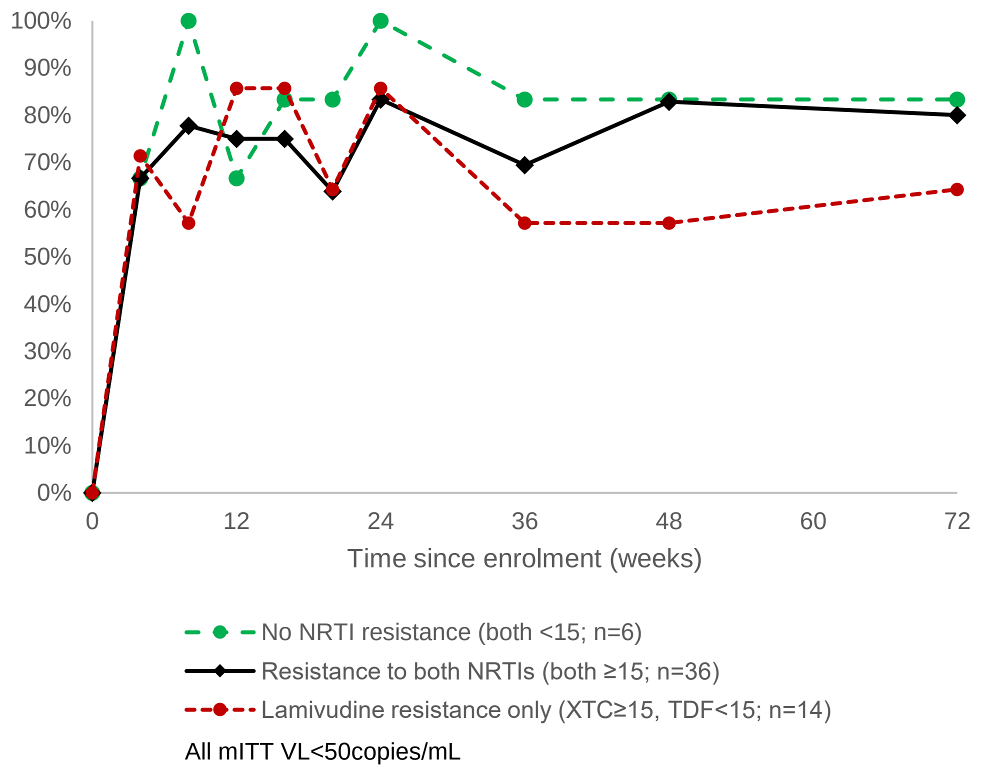

Figure 1. Proportion suppressed <50 copies/mL at each study visit in the modified intention-to-treat analysis using the Food and Drug Administration snapshot algorithm, categorised by baseline nucleoside reverse transcriptase inhibitor resistance

mITT, modified intention-to-treat; NRTI, nucleoside reverse transcriptase inhibitor; VL, viral load; XTC, lamivudine or emtricitabine.
Genotypic resistance was classified using the Stanford algorithm (version 8.9-1), with a score at least 15 indicating at least low-level resistance. Results were categorized as 6 of 56 having two fully active NRTIs (both with a Stanford score <15), 14 of 56 with resistance to lamivudine (3TC) only (tenofovir with a Stanford score <15 and XTC with a Stanford score ≥15), 0 of 56 with resistance to tenofovir only and 36 of 56 with resistance to both NRTIs (both with a Stanford score ≥15)[3].

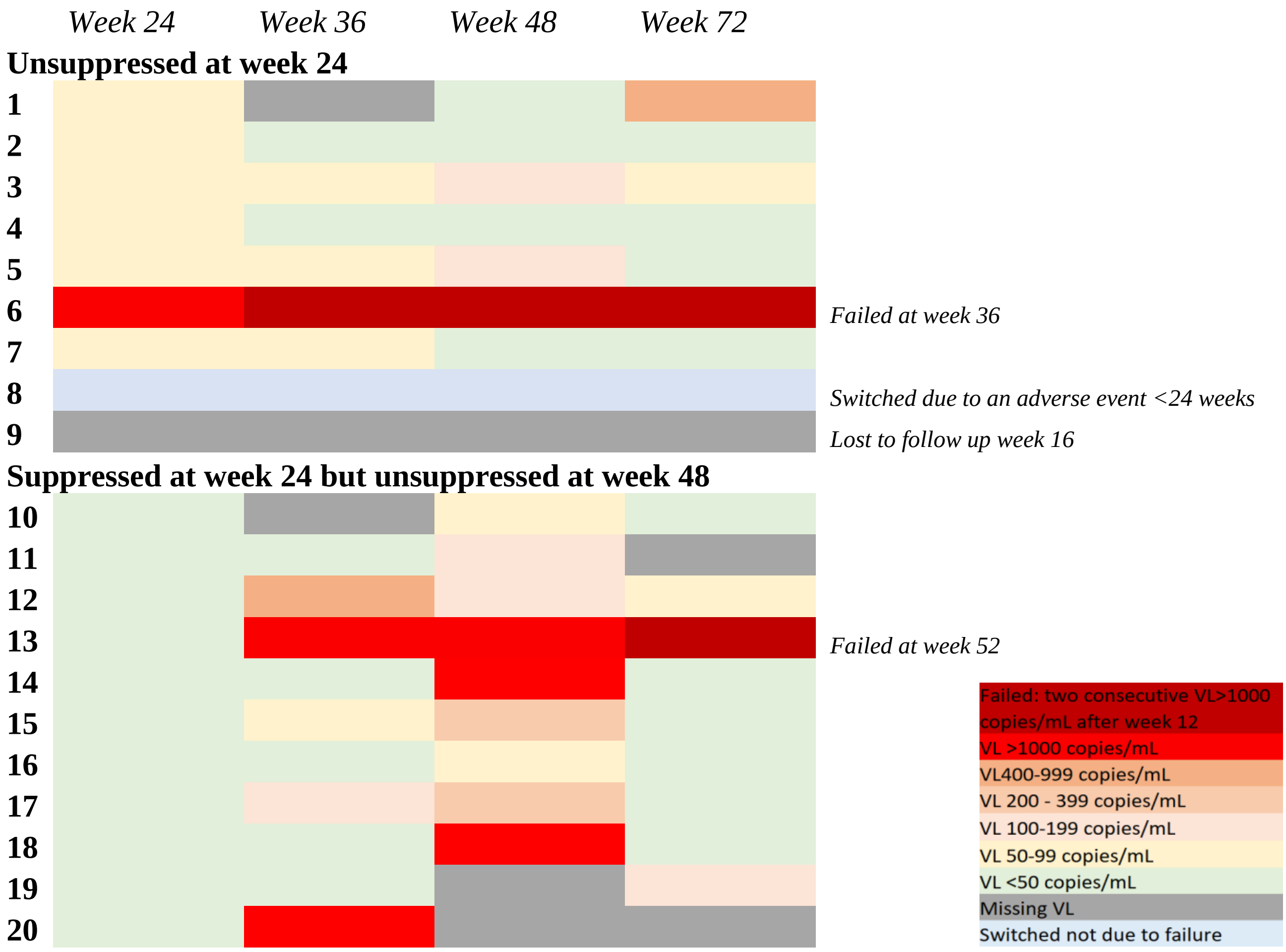

Figure 2. Virologic outcome at each time point for those not suppressed at week 24 and/or week 48
